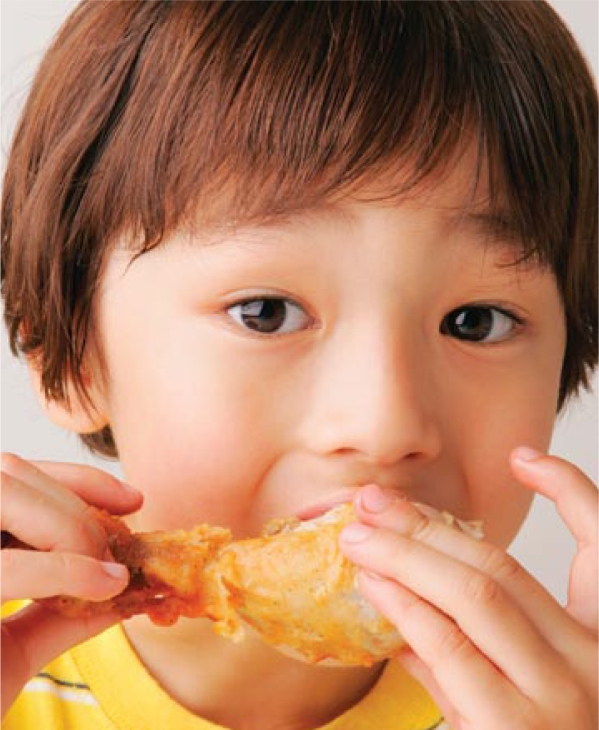# PBDEs in Diet: Meat Fat a Leading Source

**Published:** 2009-10

**Authors:** Naomi Lubick

**Affiliations:** **Naomi Lubick** is a freelance science writer based in Zürich, Switzerland, and Folsom, California. She has written for *Environmental Science & Technology*, *Nature*, and *Earth*

Although researchers have long known polybrominated diphenyl ethers (PBDEs) appear in foods such as beef, chicken, fish, and milk, the results of a new study are the first to link blood levels of prevalent PBDEs with food intake over a wide population **[*EHP*** **117:1520–1525; Fraser et al.]**. Researchers from the Boston University School of Public Health examined data from the 2003–2004 National Health and Nutrition Examination Survey (NHANES) and found that people who reported eating the most poultry and beef had higher average serum PBDE levels than people who ate lower amounts of these meats.

PBDEs are used as flame retardants for electronics, fabrics, packing materials, and other products. Some of the formulations that trigger the most concern—for example, penta‐BDE, which contains BDE‐28, ‐47, ‐99, ‐100, and ‐153—are no longer produced in Europe and the United States, but persistent PBDEs remain in such items as older electronics and upholstered furniture, and often turn up in household dust. Past surveys have found high levels of these compounds in human tissues. Fat‐soluble and persistent, PBDEs cause numerous adverse health effects in experimental animals and are suspected endocrine disruptors in humans.

The study included NHANES participants for whom there were serum measurements of up to 10 PBDE congeners as well as information on what the participants had eaten the day before the interview and their usual diet over the past year. The researchers compared the diet survey information with the results of the blood sample analyses, which had revealed detectable levels of five PBDE congeners in more than 60% of the study population.

The researchers found that, overall, vegetarians had serum PBDE concentrations approximately 25% lower than those of omnivores. The people most likely to have the highest total PBDE concentrations were young and male. Levels of all five congeners were significantly associated with high poultry fat intake. All five congeners also were associated with high intake of red meat fat, with statistically significant associations for BDE‐100, BDE‐153, and total serum PBDEs. However, the team found no significant associations between serum PBDE concentrations and high dairy or fish consumption—even though these foods also contain measurable levels of PBDEs. The authors hypothesize that although both farmed and wild fish can contain high levels of PBDEs, Americans may not consume enough fish to make this a significant source of exposure.

Next steps should include measuring food intake against BDE‐209, which is not yet included in NHANES data, and direct comparisons with exposure via dust. The authors write that researchers will also have to watch for shifts in humans’ exposure—old products will remain as reservoirs for further exposure even as manufacturers move toward alternative flame retardants, which themselves may have unexpected toxic effects.

## Figures and Tables

**Figure:**